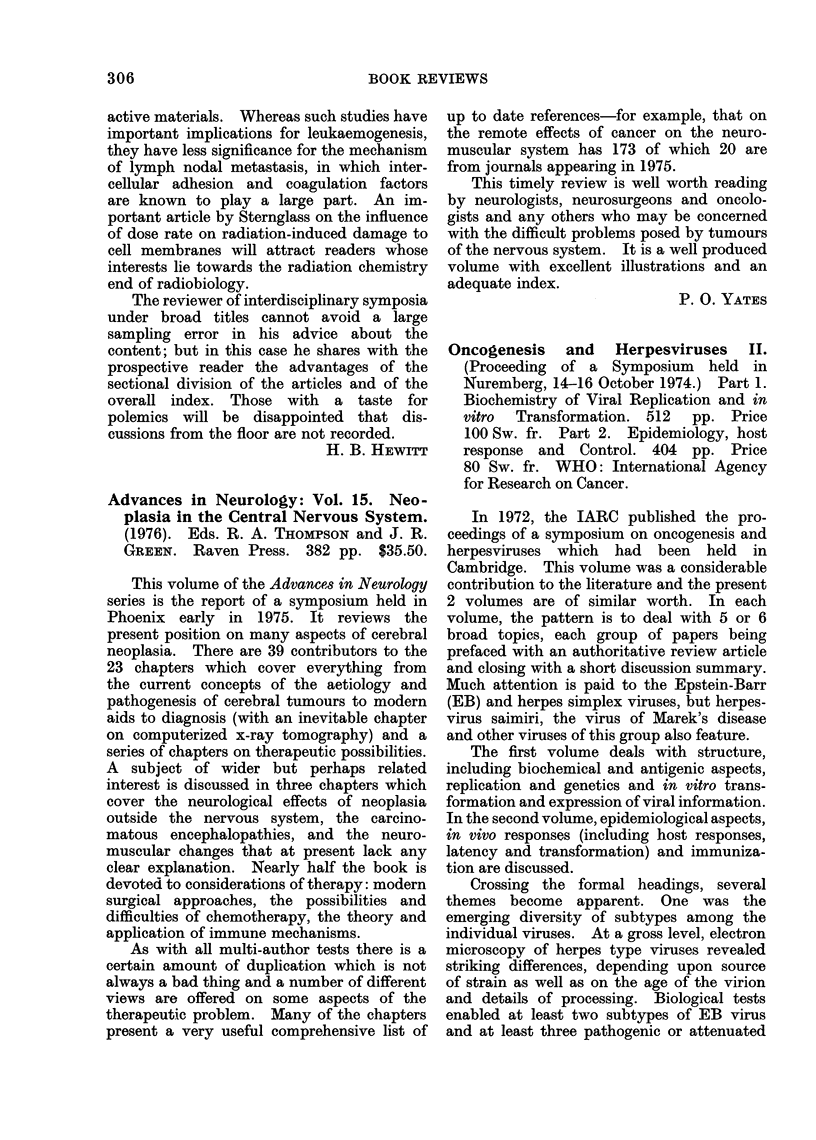# Advances in Neurology: Vol. 15. Neoplasia in the Central Nervous System

**Published:** 1976-09

**Authors:** P. O. Yates


**Advances in Neurology: Vol. 15. Neoplasia in the Central Nervous System.** (1976). Eds. R. A. THOMPSON and J. R. GREEN. Raven Press. 382 pp. $35.50.

This volume of the *Advances in Neurology* series is the report of a symposium held in Phoenix early in 1975. It reviews the present position on many aspects of cerebral neoplasia. There are 39 contributors to the 23 chapters which cover everything from the current concepts of the aetiology and pathogenesis of cerebral tumours to modern aids to diagnosis (with an inevitable chapter on computerized x-ray tomography) and a series of chapters on therapeutic possibilities. A subject of wider but perhaps related interest is discussed in three chapters which cover the neurological effects of neoplasia outside the nervous system, the carcinomatous encephalopathies, and the neuromuscular changes that at present lack any clear explanation. Nearly half the book is devoted to considerations of therapy: modern surgical approaches, the possibilities and difficulties of chemotherapy, the theory and application of immune mechanisms.

As with all multi-author tests there is a certain amount of duplication which is not always a bad thing and a number of different views are offered on some aspects of the therapeutic problem. Many of the chapters present a very useful comprehensive list of up to date references—for example, that on the remote effects of cancer on the neuromuscular system has 173 of which 20 are from journals appearing in 1975.

This timely review is well worth reading by neurologists, neurosurgeons and oncologists and any others who may be concerned with the difficult problems posed by tumours of the nervous system. It is a well produced volume with excellent illustrations and an adequate index.

P. O. YATES